# Adjuvant Radiotherapy of Involved Field versus Elective Lymph Node in Patients with Operable Esophageal Squamous Cell Cancer: A Single Institution Prospective Randomized Controlled Study

**DOI:** 10.7150/jca.50108

**Published:** 2021-03-31

**Authors:** Ruifeng Liu, Xueliang Zhang, Qiuning Zhang, Hongtao Luo, Shihong Wei, Tingting Liu, Shilong Sun, Zhiqiang Liu, Zheng Li, Jinhui Tian, Xiaohu Wang

**Affiliations:** 1 Institute of Modern Physics, Chinese Academy of Sciences, Lanzhou R.P China, 730000.; 2 Lanzhou Heavy Ion Hospital, Lanzhou R.P China, 730000.; 3 University of Chinese Academy of Sciences, Beijing R.P China, 100049.; 4 Gansu Provincial Cancer Hospital, Lanzhou R.P China, 730050.; 5 Center of Evidence Based Medicine, Lanzhou University, Lanzhou R.P China, 730000.

**Keywords:** esophageal cancer, lymph node, radiotherapy, clinical outcomes, adjuvant treatment

## Abstract

**Background:** To evaluate locoregional failure and its impact on survival by comparing involved field irradiation (IFI) with elective lymph node irradiation (ENI) for patients with esophageal squamous cell cancer who underwent post-operative radiotherapy.

**Methods and Materials:** The enrolled patients were randomized allocated to IFI or ENI group. CTV of IFI was generated according to pre-operative primary tumor location and post-operative pathological characters and positive LNs regions. CTV of ENI was generated according to pre-operative tumor position to administer selective lymph node irradiation. Radiotherapy planning was delivered using either 3D-CRT or IMRT.

**Results:** A total of 57 patients were enrolled, 28 patients in ENI group and 29 patients in IFI group. There were not statistical differences between two groups in baseline (*p*>0.05). The initial locoregional failure rate was 17.9 % in ENI arm and 20.7% in IFI arm respectively (*p*=0.085). The 1-, 3-, and 5-year Progression-free Survival (PFS) were 63.2, 43.5, and 21.8 % in ENI arm versus 78.2, 60.1, and 55.1% in IFI arm (*p* =0.038). The 1-, 3-, and 5-year overall survival (OS) were 78.6, 46.9, and 23.5 % in ENI arm versus 72.9, 59.7, and 54.3 % in IFI arm (*p*=0.06). Acute radiation pneumonitis (*p*=0.005) and hematological toxicities (*p* =0.029) also showed statistical differences between groups, ENI arm was more than IFI arm.

**Conclusions:** The results indicated that IFI tended to improve survival and reduce toxicities for patients with operative ESCC and did not increase locoregional failure compared to ENI. It is thus suggested that IFI for ESCC PORT is worthy of clinical recommendation and further study.

## Introduction

Esophageal cancer (EC) is the 8^th^ most common cancer worldwide. In China, an estimated 320,800 new cases resulted in 253,800 deaths from the disease in 2018 1,2. Esophageal squamous cell carcinoma (ESCC) is the main histological subtype in Asian countries. However, in recent decades, the incidence of esophageal adenocarcinoma (EAC) has been increasing in the West 3,4,5. Although surgery is currently the mainstay of treatment in the early stage ESCC and as an important component of multimodality treatment in locally advanced ESCC, it exhibits modest efficacy, with a 5-year survival rate of less than 30% 6. Approximately half of patients present with locally advanced or metastatic disease at initial diagnosis, and over a third develop regional recurrences or distant metastases after surgery. These results have led to the development of multimodality combination therapy, which includes surgery, chemotherapy, and radiotherapy.

The current National Comprehensive Cancer Network (NCCN) guidelines recommend neoadjuvant or definitive chemoradiation for locally advanced EC 7. However, these recommendations are often not implemented in clinical practice. In patients with locally advanced ESCC, curative surgery is usually performed at initial diagnosis rather than after neoadjuvant chemoradiotherapy. These patients need to undergo subsequent adjuvant radiotherapy in order to reduce locoregional recurrence and improve progression-free survival (PFS) and overall survival (OS). A review of pertinent literature reveals reports on several non-randomized trials, some of which have evaluated the survival benefit of post-operative radiotherapy (PORT) with inconsistent results. There has been much debate with respect to pN0 patients, many early studies reported a lack of clinical benefit. However, many recent studies concluded that it was beneficial to pN0 patients 8,9,10. Sure, the value of PORT is established in patients with pathologically confirmed positive lymph nodes 11. One of the major reasons for the controversy on clinical benefit was the lack of consensus on the design of an optimal radiation target volume. In cases of locally advanced EC, the use of radical involved field irradiation (IFI) has become increasingly popular among radiation oncologists. Compared to elective nodal irradiation (ENI), nodal failure rates are not higher with IFI, but esophageal toxicities are lower 12. A meta-analysis including 10 studies with a total of 1348 patients compared the relative advantages and disadvantages of ENI and IFI for the definitive radiotherapy of ESCC. No significant differences in local control or OS were noted between ENI and IFI, and the incidences of ≥grade 3 acute esophagitis and pneumonitis were significantly lower in the IFI group. In addition, the use of IFI did not increase the incidence of out-of-field recurrences or metastases. These results generated interest in the use of IFI for PORT in ESCC 13,14.

Up to now, no studies have compared post-operative IFI and ENI in patients with operable ESCC. To address this issue, we performed a preliminary prospective single center randomized controlled study on PORT to investigate the advantages and disadvantages of using IFI and ENI on survival and toxicities in ESCC. The findings are expected to provide guidance for clinical practice.

## Materials and Methods

### Patient population

From January 2012 to December 2018, we conducted a randomized controlled trial in the Gansu Provincial Cancer Hospital to compare IFI and ENI in patients undergoing PORT for ESCC. The inclusion criteria was as follows: 1) patients older than 18 at diagnosis, 2) Karnofsky performance status (KPS) score ≥70, 3) pathologically confirmed ESCC, 4) surgical resection followed by radiotherapy (RT) or chemoradiotherapy (CRT), 5) R1 or R2 resection with any stage, node positive or pT2-4a, 6) radiotherapy delivered using either three-dimensional conformal radiotherapy (3D-CRT) or intensity modulated radiation therapy (IMRT) techniques. The exclusion criteria were as follows: 1) non-ESCC patients, 2) recurrence or metastasis prior to radiotherapy, 3) incomplete RT schedules, and 4) incomplete data on outcomes. Prior to patient enrollment, the study had been approved by the ethics committee of the Gansu Provincial Cancer Hospital (Approval number: A2013112260042). Randomization was performed by computer generated random numbers before initiation of the trial in January 2012. Allocation concealment was implemented on the patients after informed consent. Sample size calculation was based on a margin of non-inferiority for median relapse-free survival time was 11 months that was estimated from previous studies 15. We found that at least 57 subjects were required in each group in order to obtain a power of 90% and a two-sided α-level of 0.05 to demonstrate the non-inferiority of IFI with ENI. However, in view of the slow enrollment process, only total 60 subjects were enrolled from January 2012 to June 2018, 3 of including patients with discontinued radiotherapy after enrollment. Finally, 57 patients with complete data, including 28 and 29 patients in the IFI and ENI groups, respectively, were analyzed.

### Radiotherapy target volume in patients undergoing ENI

In patients undergoing ENI, the clinical target volume (CTV) was generated according to the pre-operative tumor position to administer selective node irradiation.

#### CTV of the upper thoracic segment

The upper boundary was at the level of cricothyroid membrane, and the lower boundary was at a level of 3 cm below the tracheal carina. Generally, the target volume incorporates the corresponding areas of lymphatic drainage, the anastomosis, and the primary esophageal tumor bed. The inferior cervical, supraclavicular, and mediastinal areas 2, 4, and 7 lymph node (LN) regions should be included.

#### CTV of the middle thoracic segment

The upper boundary was at the level of cricothyroid membrane, and the lower boundary was at a level of 3-5 cm below the preoperative tumor bed. In practice, the target volume should include the corresponding areas of lymphatic drainage, the anastomosis, and the primary esophageal tumor bed. Therefore, bilateral cervical, para-esophageal, lower cervical, supraclavicular, and mediastinal areas 2, 4, 7, and 8 LN regions should be included.

#### CTV of the lower thoracic segment

The upper boundary was at the level of the thoracic inlet, and the lower boundary was at a level of 3-5 cm below the tumor bed. The delineation of the target volume should include the corresponding areas of lymphatic drainage, the anastomosis, and the primary esophageal tumor bed. Therefore, the mediastinal areas 2, 4, 7, and 8 LN regions should be included, but the paracardial and left gastric arterial LN regions are not included unless they are either pathologically positive or enlarged on computed tomography (CT) images.

**Figure 1:**
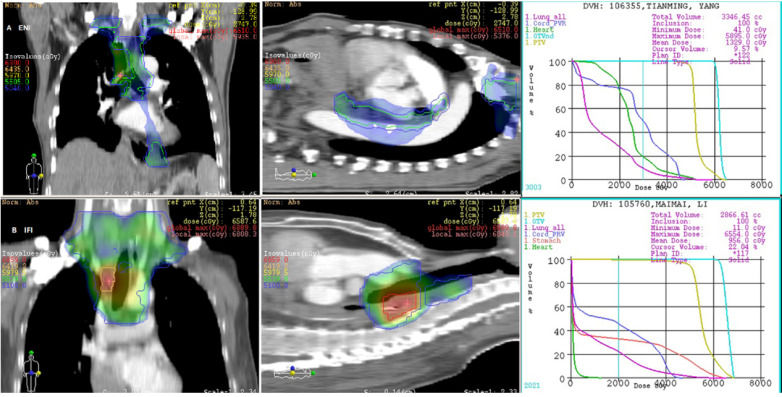
Irradiation field of postoperative radiotherapy using ENI or IFI for 2 patients with upper thoracic esophageal carcinoma.

The LN gross tumor volume (GTVn) was defined as the mediastinal LNs with a diameter of at least 1 cm in the short axis, or more than 5 mm in the short axis in the paraesophageal region, tracheoesophageal sulcus, and diaphragmatic angle. Multiple clusters of LNs on the CT image, areas of increased uptake on fluorodeoxyglucose-positron emission tomography (FDG-PET) (excluding physiological accumulation), and pathologically confirmed residual disease were also included in the GTV. The planning target volume (PTV) was then generated by applying 5mm radial and 10 mm longitudinal margins to the CTV, respectively. In those undergoing ENI, a dose of 50-50.4 Gy in 1.8-2 Gy per fraction over 5-5.6 weeks was delivered to the CTV. The GTVs were boosted to a dose of 60 Gy (see Fig. 1A).

### Radiotherapy target volume in patients undergoing IFI

The CTV was delineated based on the pre-operative location of the primary tumor and the post-operative extent of pathological invasion, including positive LN regions. Radial and longitudinal margins of 5 and 20 mm, respectively, were added to the tumor bed and surrounding area of lymphatic drainage; positive LN regions also were included regardless of the location of the primary tumor. Anastomoses were not included unless the margins were positive. The GTVn was similar to that of the ENI group. The PTV was generated by applying 5mm radial and 10 mm longitudinal margins to the CTV. Patients in the IFI arm received a dose of 50-50.4 Gy, delivered in 1.8-2 Gy per fraction over 5-5.6 weeks. The GTV was boosted to at least 60 Gy. The mediastinal LN regions were not electively irradiated in this group (see Fig. 1B).

### Treatment planning evaluation and organ-at-risk (OAR) limitations

Radiotherapy planning was delivered with 6-10 MV photons using Elekta Synergy linear accelerator with either 3D-CRT or IMRT precision radiotherapy technique. The dose homogeneity criteria ensured that the respective PTV-min exceeded 90% of the prescribed dose, and PTV more than 110% of the prescribed dose was below 5%. The mean lung dose (MLD) was restricted at or below 15 Gy; in terms of lung volume doses, V20 (the lung volume receiving over 20 Gy) <30% or V30 <20% was maintained. Maximum dose of spinal cord was restricted at or below 45 Gy. The maximum dose to the intrathoracic stomach was restricted to below 60 Gy; alternatively, V40 <50% was maintained 16.

### Chemotherapy

Neoadjuvant chemotherapy regimens were not specified before enrollment. However, subgroup analysis was performed while summarizing data. Concomitant chemotherapy was not administered during adjuvant radiotherapy. All patients received 2-4 cycles of adjuvant chemotherapy following completion of RT. Chemotherapy regimens comprised fluorouracil (5-FU) or docetaxel (TXT) doublets including either cisplatin (DDP) or nedaplatin (NDP). In patients aged 75 years or older, dose reductions to 80% of the dose were made. Adjuvant chemotherapy was initiated 2-4 weeks after either surgery or PORT.

### Data collection

The primary endpoints were the initial locoregional failure rate and the incidence of toxicity. The secondary endpoints were PFS, OS, and dosimetry parameters. The variables collected for each patient included baseline characteristics (age and sex), pre-radiotherapy KPS score, characteristics of the tumor (primary site, extent of lesion, stage of tumor, histology, grade, and nodal status), type of surgery, status of surgical margins, interval between surgery and RT, extent of irradiation field, RT technique, RT dosimetry parameters (total RT dose, target volume, and OAR dose), chemotherapy agents, chemotherapy cycles, date of recurrence or progression, and date of death. All the relevant data were obtained from the hospital records.

### Statistical analyses

The constituent ratio was analyzed by the chi-square test, and the measurement data were analyzed by the nonparametric test. The Kaplan-Meier method with the log-rank test was employed to estimate differences in 1-, 3-, and 5-year PFS and OS between patients receiving IFI and ENI. The t-test was used to compare the dosimetry differences. All *p* values were based on a 2-sided test, and the differences were regarded as statistically significant when *p*<0.05. All statistical analyses were performed using the SPSS (Version 22.0) statistical software package.

### Follow-up and evaluation criteria

All patients who completed treatment were routinely followed-up every 3 months in the first 2 years, every 6 months in the third to fifth year, and every 12 months thereafter. Follow-up routinely included physical examination, laboratory examination, CT scans of the thorax and upper abdomen, and ultrasound examination of the cervical LNs. Upper gastrointestinal endoscopy (with or without biopsy) was not performed unless indicated. CT scan of the thorax and upper abdomen was performed at each visit, and PET/CT was performed every 6-12 months or in cases of suspected recurrence.

## Results

### Patient characteristics

Till date, 57 patients with ESCC who underwent esophagectomy have been enrolled in this trial. The characteristics of the 57 eligible patients are shown in Table 1. Gender, age, performance status, cancer staging, tumor extent and location, surgical procedures, LN dissection rate, RT techniques, interval between surgery and RT, and cycles of neoadjuvant chemotherapy were not statistical differences between two groups (*p*>0.05). However, the cycles of adjuvant chemotherapy differed significantly between the two groups (*p*=0.031) (see Table 1).

### Locoregional failure, distant metastases, and survival

Locoregional failure includes recurrence at the site of the primary tumor and regional LN progression. Overall, 5 (17.9%) and 6 (20.7%) patients in the ENI and IFI groups had initial locoregional failure, respectively (*p*=0.085). Among them, 4 (14.3%) and 2 (6.9%) patients experienced recurrence of the primary tumor, while 1 (3.6%) and 4 (13.8%) patients experienced progression in the regional LNs in the ENI and IFI groups, respectively. Initial distant metastases were observed in 7 (25%) and 7 (24.1%) patients in the ENI and IFI groups, respectively (see Table 2).

**Figure 2:**
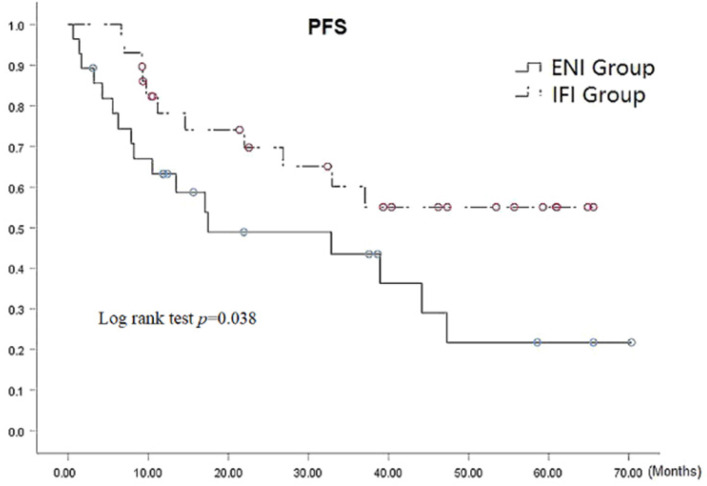
PFS Curves for patients with ENI vs. IFI.

At the time of analysis, 29 patients, comprising 11 (39.3%) and 18 (62.1%) patients in the ENI and IFI groups, respectively, were alive. It should be noted that there is very small statistical difference was observed between the groups (*p*=0.085). The respective 1-, 3-, and 5-year PFS rates were 63.2%, 43.5%, and 21.8 % in ENI group and 78.2%, 60.1%, and 55.1 % in IFI group, (*p*=0.038 by the log-rank test) as shown in Fig. 2, there was statistical difference in 5-year PFS (*p*=0.009). The median PFS was 17.5 (95% CI: 5.3-42.9) months versus 37.1 (95% CI: 19.2-54.9) months in the ENI and IFI groups, respectively. Furthermore, the PFS was significantly superior in the IFI group than that of the ENI group.

**Figure 3:**
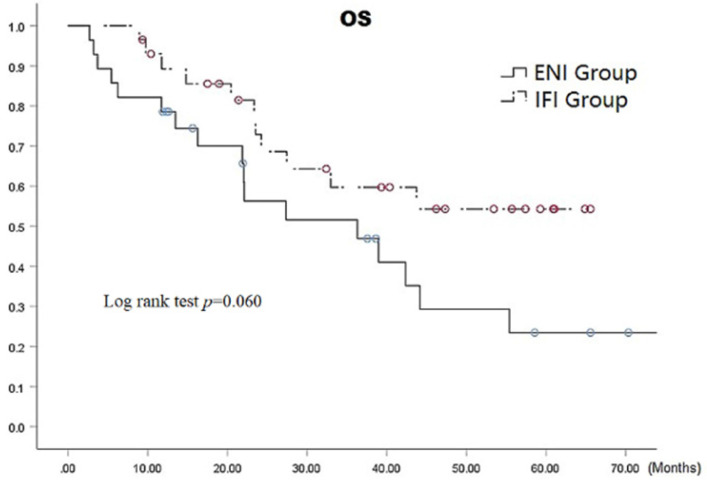
OS with Stage Curves for all patients.

**Figure 4:**
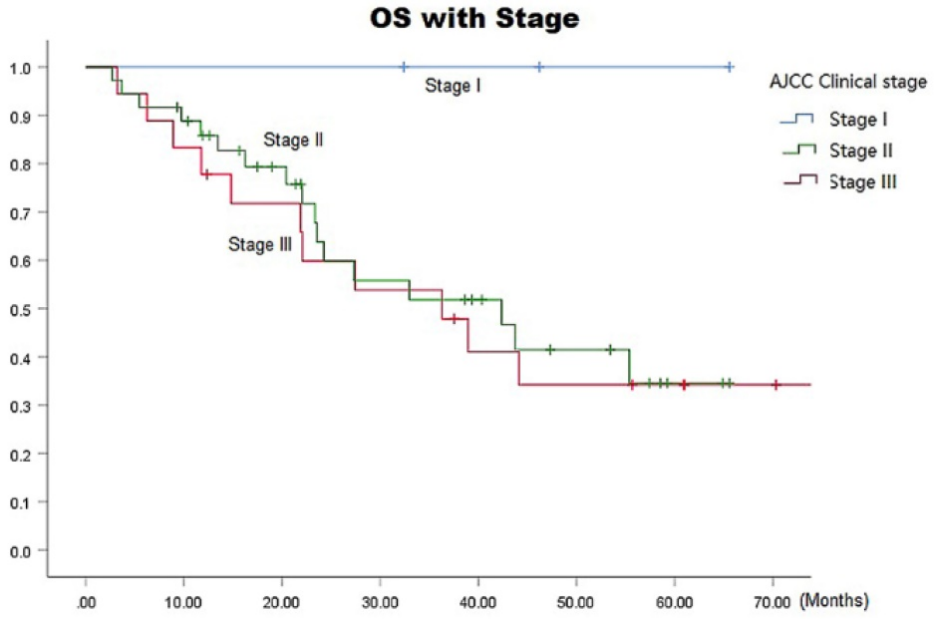
OS Curves for patients with ENI vs. IFI.

The median survival time in the ENI and IFI groups was 36.3 (95% CI: 13.8-58.9) months versus 42.4 (95% CI: 30.2-54.6) months, respectively. The respective 1-, 3-, and 5-year overall survival (OS) rates in the ENI and IFI groups were 78.6%, 46.9%, and 23.5% versus 72.9%, 59.7%, and 54.3 (*p*=0.06 by the log-rank test), as shown in Fig. 3, there was statistically difference in 5-year OS (*p*=0.020). The respective overall 1-, 3-, and 5-year OS rates were 100% each in stage I, 85.8%, 51.8%, and 34.6% in stage II, and 77.8%, 47.9%, and 34.2% in stage III, respectively (*p*= 0.0001) (Fig. 4).

### Acute and late toxicities

Acute and late radiation induced toxicities were evaluated based on the Radiation Therapy Oncology Group (RTOG) toxicity criteria 17. Wilcoxon rank sum tests were used to compare the incidence of toxicities between the two groups, by grade. The incidences of acute radiation pneumonitis (*p*=0.005) and hematological toxicities (*p*=0.029) were significantly higher in the ENI group than in those receiving IFI. No statistical differences were observed in terms of acute radiation-induced esophagitis (*p*=0.344), acute digestive toxicities (*p*=0.428), late esophageal stenosis (*p*=0.110), pulmonary interstitial fibrosis, (*p*=0.055), and late heart injury (*p*=0.309) between two groups. We compared toxicities of grades 3-5 as severe toxicity using the Chi-square test. Higher than grade 3 acute radiation-induced esophagitis, acute radiation pneumonitis, hematological toxicities, acute digestive toxicities, late esophageal injury, and late lung injury were observed in 4 (10.3%), 1 (3.6%), 8 (28.6%), 1 (3.6%) and 1 (3.6%) patients, respectively, in the ENI group, and in 1 (3.4%), 1 (3.4%), 3 (10.3%), 1 (3.4%) and 1 (3.4%) patients in the IFI group (see Table 3). Notably, all 3 patients with grade 5 toxicity in the ENI group had following issues, who died of esophageal perforation, upper gastrointestinal bleeding, and esophagotracheal fistula, respectively.

### Dosimetric evaluation

Dose volume histograms were used to calculate the dosimetry parameters. There were statistical differences between the groups in terms of PTV (*p*=0.014), GTV (*p*=0.038), maximum dose of spinal cord (*p*=0.024), and MLD (*p*=0.049), V5 (*p* =0.005), V10 (*p=*0.042), V20 (*p*=0.018), V30 (*p* =0.035) of the lungs, the ENI group demonstrated higher values than the IFI group. No statistical differences were noted between the two groups in terms of mean PTV and GTV doses, maximum dose to the intrathoracic stomach, and V30 of the heart; *p* >0.05 (see Table 4).

## Discussion

This study aimed to compare the differences in locoregional failure, survival, toxicities and dosimetry between ENI and IFI for patients receiving PORT with operable ESCC. Since it was proposed in the 1960s, the value of PORT in EC has been widely reported. A large randomized controlled clinical trial from China further confirmed the value of PORT in ESCC 18. In that study, 495 patients who has been through the radical surgery for ESCC were randomized allocated by the envelope method to receive surgery (n=275) or surgery with radiotherapy (n=220); the findings showed that prophylactic PORT improved survival in patients with stage III tumors who underwent radical resection (5-year OS: 13.1% vs. 35.1%, respectively) and reduced the incidence of failure in the intra-thoracic LNs and anastomosis (19.8% vs. 44.9%), without any increase in the incidence of anastomotic strictures. Nevertheless, many earlier randomized controlled trials demonstrated inconsistent results. For instance, Fok et al. and Ténière et al. reported no difference in survival among patients receiving PORT or surgery alone 19,20. However, in interpreting these findings, it is important to note that the aforementioned results in the era of two-dimensional RT planning, when treatment was delivered using non-conformal anteroposterior-posteroanterior fields. Advanced RT techniques had not emerged, and the treatment fields were usually more extensive. Therefore, these findings from the early 1990s are not representative of current treatment outcomes. It was therefore essential to re-evaluate the influence of PORT fields on OS and local-regional recurrence in the era of precision RT. Although there are some studies on the extent of RT fields, most of them have focused on radical radiotherapy instead of PORT 21. Till date, two meta-analyses have compared IFI with ENI for radical CRT in EC. The results have shown no significant differences between those receiving IFI and ENI, in terms of OS and local control 22,23. However, the incidence of esophageal and lung toxicities was significantly lower in those receiving IFI. These results show that IFI is a viable treatment option in locally advanced EC, and is particularly useful in minimizing radiation-related toxicities. To the best of our knowledge, no prospective studies have compared ENI with IFI in patients receiving PORT for EC.

There is currently no consensus on the optimal CTV extent for PORT in EC. Usually, the target volumes for PORT depend on the following factors: 1) stage and location of the primary lesion; 2) major sites of relapse; 3) and the area of surgical non-clearance. However, owing to nodal skip metastases and inconsistencies in surgical procedures, it is often difficult to evaluate the exact major sites of relapse, and the areas of surgical non-clearance. Therefore, the NCCN guidelines suggest that the standard PORT CTV should encompass the tumor bed, bilateral supraclavicular LN areas, mediastinal LNs, and the LNs around the cardia and left gastric artery 8. It appears that the guideline has ignored the individual characteristics of patients with ESCC. Since the recommended extent of the PORT surpass that of radical radiotherapy, this issue deserves serious consideration. Some studies found the probability of skip metastases in EC to be only 5%-10% 17,24. This may allow scope for optimization of irradiation fields to reduce adverse events. Previous studies have provided several suggestions for the extent of the CTV of PORT in ESCC. These include: 1) bilateral supraclavicular areas from the level of the Adam's apple to the subclavian artery, the entire mediastinum, and the left gastric lymphatic area 17,25, 2) bilateral supraclavicular regions, entire mediastinum, and the left gastric region only if it was involved 20, 3) tumor bed only, and bilateral supraclavicular and left gastric lymphatic areas for lesions in the upper and lower thoracic segments, respectively 26, 4) margins of 3-4 cm around the tumor bed in the cranio-caudal direction, without inclusion of left gastric and bilateral supraclavicular areas for prophylactic irradiation 27, 5) T-shaped fields including bilateral supraclavicular and upper mediastinal areas 28. Despite many attempts to minimize the PORT field, the exact coverage of high-risk areas was difficult to achieve with two-dimensional radiotherapy.

The study has found that the incidence of local lymph node metastasis is related to the site of the primary tumor 29. For example, upper thoracic esophagus tumor often metastasizes to cervical lymph nodes, while lower thoracic tumor are more likely to metastasizes to upper abdominal lymph nodes. Therefore, there may be a special relationship between the tumor location and the recurrence of LN. In view of these findings, ENI fields were usually the most widely accepted for adequate CTV coverage for either radical radiotherapy or PORT. However, the use of IFI has become increasingly popular in the treatment of locally advanced EC. In IFI, the CTV is usually generated by adding no radial margins and but only adding 2 cm longitudinal margins to the GTV-primary; no margins are added for the GTV-LNs, and the mediastinal LNs are not electively irradiated, only involved LN areas, diagnosed by imaging, are irradiated 23,24. This method of accurate CTV delineation was made possible by the advent of precision radiotherapy and by improvements in imaging. IMRT involves computer-based planning; it has improved target volume coverage in EC with less off-target delivery, potentially reducing toxicities and allowing further dose escalation beyond the limits of 3D-CRT 30. Therefore, if advanced radiotherapy techniques and imaging are employed, IFI may be feasible for PORT in ESCC. In this study, we re-defined the PORT fields as suggested by the expert consensus and recommendations for target volume delineation in the NCCN guideline 8,28,31,32,33. In our study, the CTV for IFI was generated according to the pre-operative location of the primary tumor and the post-operative extent of pathological invasion and positive LNs regions. The CTV included 5 mm radial and 20 mm longitudinal margins, respectively, to the tumor bed and positive LN regions; the anastomosis was not included unless the margin was positive. The implementation of this PORT field necessitates the use of standardized surgical procedures for radical esophagectomy. These include either a three- or two- field lymphadenectomy, since recent studies have demonstrated no obvious differences in impact of either approach on the PORT CTV 34,35.

In the current study, the 3- and 5-year OS were 59.7% and 54.3% in IFI group versus 46.9% and 23.5% in ENI group, respectively. Although the difference in OS between the groups was not statistically significant (*p*=0.06), the absolute survival time in the IFI group was longer. Moreover, the 3- and 5-year PFS were 60.1% and 55.1% in IFI group versus 43.5% and 21.8% in ENI group, respectively (*p*=0.038). These results were evidently superior to that of previous reports from randomized trials on ENI. Chen et al. 36 reported a 3- and 5-year OS of 53.1% and 44.6%, respectively, using T-shaped fields to treat node positive thoracic ESCC in patients who underwent 3-field lymphadenectomy with esophagectomy. Xiao et al. 37 randomized patients with ESCC who underwent radical esophagectomy to receive surgery alone or PORT; the CTV included bilateral supraclavicular regions, the whole mediastinum, and the left gastric arterial LN region. The 5-year OS was 41% with acceptable toxicities. Wang et al. 38 conducted a randomized controlled clinical trial to explore whether PORT could improve the prognosis in patients with ESCC who were at a high risk of poor clinical outcomes; the 5-year OS was 48.1%. Considering the possibility of longitudinal drainage of submucosal lymphatic network and skip lymph node metastasis in ESCC, the standard PORT CTV should include tumor bed, bilateral supraclavicular region, mediastinal LNs, cardia and left gastric region 39,40. Till date, few studies have employed the exact standard radiation field without suitable adjustments, as serious complications may nullify the survival advantage in certain patients. In our current study, the incidence of acute and late grade 3-5 adverse events in the IFI group was quite low, and there were no fatal toxicities. In addition, the incidence of more than grade 3 acute radiation-induced esophagitis, acute radiation pneumonitis, hematological toxicities, acute digestive toxicities, late esophageal injury and late lung injury only all was only 3.4% in all patients. Conversely, in the ENI group, the incidence of grade 3-5 acute and late radiation related adverse events were higher, with 3 related deaths. Among them, 1 patient each died of acute esophageal perforation, acute upper gastrointestinal bleeding, and a late esophageal mediastinal fistula, respectively.

The rates of initial locoregional failure and distant metastases were similar in both groups. Notably, till the date of last follow-up, compared to 1 patient in the ENI group, 4 patients in the IFI group had regional LN recurrences. The sites of failure were the commonly reported sites, viz., bilateral supraclavicular areas and the superior mediastinum. The findings suggest that regional lymph node have a high-risk of failure rate in IFI group, particularly in the supraclavicular and the superior mediastinal areas. Further large sample controlled clinical trials are necessary for validation, we should be careful in final conclusion. However, the rates of distant metastases were similar in both groups.

Dosimetry parameter is a very important issue of concern for radiation oncologists. Doses of OARs are of particular interest as these are related to clinical advantages and disadvantages. It is obvious that any reduction of the target volume leads to reductions in the doses of OARs, with consequent lowering of radiation toxicities. We had explored various physics-based improvements for radiotherapy of the entire esophagus and T-shaped field using IMRT. The results had demonstrated that small field IMRT plans provided superior lung and heart sparing compared to larger IMRT fields; the jaw tracking technique provided further normal tissue sparing compared to fixed jaw plans 41,42,43. In the current study, there were statistical differences between the fields in terms of may parameters including PTV, GTV, MLD, V5, V10, V20, and V30 of the lungs, and the maximum dose to the spinal cord. All of these were higher in the ENI group.

Although preoperative chemoradiotherapy is being increasingly recommended as a preferred treatment model for locally advanced EC, the prevalence and availability of this approach is a matter of concern 44,45. Most surgeons prefer either preoperative chemotherapy or surgery alone instead of neoadjuvant chemoradiotherapy; this makes post-operative chemoradiotherapy essential for stage II or III ESCC. Sadrizadeh A. et al. compared the benefits of neoadjuvant chemoradiotherapy with that of postoperative chemoradiotherapy (POCRT). The results indicated that neoadjuvant chemoradiotherapy offered no survival advantage over POCRT. However, POCRT was associated with a higher risk of postoperative complications 46. In the current study, adjuvant chemotherapy was administered to 85.7% and 75.8% in ENI and IFI groups, respectively; the number of chemotherapy cycles were higher in ENI group than that of IFI group (3.1 vs. 2.4); this also could explain the higher incidence of toxicities in the former. In view of the risk of cumulative toxicities from either treatment modality, the PORT field should be delineated with care in cases of POCRT. Several studies have demonstrated that in ESCC, POCRT is significantly more effective than PORT alone. This is particularly applicable for patients with vascular emboli and other poor prognostic factors 47. Nevertheless, postoperative chemotherapy could safely be added to PORT either using ENI or IFI, and POCRT plays an increasingly important role in the comprehensive treatment of EC.

The current prospective clinical trial has certain limitations. The findings in this study may have been biased owing to significant differences in T and N stages, and performance status, between the groups. The IFI group demonstrated superior survival with more patients with T1 and N1 stages, and good performance status. More patients with N2-3 disease and inferior performance status were in the ENI arm. These may have affected the data on treatment outcomes. In addition, PET-CT was not routinely used in defining viable lymph node metastases; this may have affected the accuracy of target volume delineation. Moreover, multiple factors such as tumor location, POCRT, and TNM stage, among others, influence postoperative recurrence in ESCC.

## Conclusion

In summary, compared to ENI, IFI provided superior survival in this cohort, with less toxicities in patients receiving PORT for ESCC. In addition, IFI did not increase locoregional failure. IFI may be recommended in the clinical practice for PORT in ESCC. Further studies are needed to validate our findings.

## Figures and Tables

**Table 1:** Patients' clinical characteristic

Factor	ENI n (%)	IFI n (%)	χ^2^ (*F value)	P value
**Total**				
Subgroup	28	29		
**Sex**				
Female	2 (3.5)	1 (1.7)	0.390	0.532
Male	26 (45.6)	28 (49.2)		
**Age (y.o.)**				
Range	40-69	46-76	0.016*	0.900
Median	58.5±8.7	58.7±8.6		
**ECOG**				
1	8 (14.0)	13 (22.8)	3.292	0.193
2	18 (31.6)	16 (28.1)		
3	2 (3.5)	0		
**Location**				
Ut	2 (3.5)	4 (7.0%)	0.697	0.706
Mt	15 (26.3)	15 (26.3)		
Lt	11 (19.4)	10 (17.5)		
**Lesion length (cm)**				
Range	3-10	3-7	1.524*	0.222
Median	5.3±1.6	4.5±1.1		
**Surgical procedure**				
Single incision	15 (26.3)	13 (22.8)	3.656	0.161
Two incisions	10 (17.5)	7 (12.3)		
Three incisions	3 (5.3)	9 (15.8)		
**Clinical stage**				
I	0	3 (5.3)	5.130	0.163
II	11 (19.3)	7 (12.3)		
III	17 (29.8)	19 (33.3)		
**Lymph node dissection**				
No. of positive	2.5±2.9	2.3±3.7	0.229*	0.634
No. of total	16.5±8.5	14.8±8.8	0.000*	0.997
**RT technology**				
3D-CRT	7 (12.3)	10 (17.5)	0.612	0.434
IMRT	21 (36.8)	19 (33.3)		
**Interval between surgery to RT (mo)**				
Range	0.9-10	0.6-10.9	0.221*	0.640
Median	3.9±1.9	2.7±1.9		
**Pts of neoadjuvant chemo**	4 (14.3)	6 (20.7)		
No. of cycles	1.7±0.5	2.3±1.9	1.610*	0.240
**Pts of adjuvant chemo**	24 (85.7)	22 (75.9)		
No. of cycles	3.1±1.4	2.4±1.0	4.995*	0.031
**Follow-up time(mo)**				
Range	2.7-78.8	8.9-65.6	0.185*	0.669
Median	28.5±21.7	34.6±19.2		

Note: *F value in T test. Abbreviations: ENI, elective lymph node irradiation; IFI, involved field irradiation; y.o., years old; mo, months; RT, radiotherapy; Pts, patients; Chemo, chemotherapy; No., numbers.

**Table 2:** Treatment results between ENI and IFI

Factors	ENI n (%)	IFI n (%)	χ^2^	*P* value
**Total**				
Subgroup	28	29		
**State at analysis**				
Alive	11 (39.3)	18 (62.1)	2.959	0.085
Dead	17 (60.7)	11 (37.9)		
**Cause of death**				
Cancer related	12 (42.9)	8 (28.5)	0.15	0.903
Non-cancer related	5 (17.9)	3 (10.7)		
**First locoregional failure**	5 (17.9)	6 (20.7)	0.073	0.786
Primary tumor	4 (14.3)	2 (6.9)		
Regional lymph node	1 (3.6)	4 (13.8)		
**First distance metastasis**	7 (25.0)	7 (24.1)	0.006	0.940
Lymph node	2 (7.1)	3 (10.7)		
Lung	2 (7.1)	2 (6.9)		
Liver	1 (3.6)	1 (3.4)		
Bone	2 (7.2)	1 (3.4)		
**PFS**				
1-year	18 (63.2)	23 (78.2)	1.593	0.207
3-year	12 (43.5)	17 (60.1)	1.416	0.234
5-year	6 (21.8)	16 (55.1)	6.844	0.009
**OS**				
1-year	22 (78.6)	21 (72.9%)	0.292	0.589
3-year	13 (46.9)	17 (59.7%)	0.849	0.357
5-year	7 (23.5)	16 (54.3%)	5.388	0.020

Abbreviation: ENI, elective lymph node irradiation; IFI, involved field irradiation; OS, overall survival; PFS, progress-free survival.

**Table 3:** Comparison of grade 3-5 acute and late radiated adverse events between two groups

Factors	Grade	ENI (n%)	IFI (n%)	X^2^	*P* value
Acute radiation-induced esophagitis	Grade 3	2 (7.1%)	0 (0%)	2.091	0.148
	Grade 4	1 (3.6%)	1 (3.4%)		
	Grade 5	1 (3.6%)	0		
Acute radiation pneumonitis	Grade 3	1 (3.6%)	1 (3.4%)	0.001	0.980
Hematological toxicities	Grade 3	8 (28.6%)	3 (10.3%)	3.039	0.081
Acute digestive toxicities	Grade 3	2 (7.1%)	1 (3.4%)	1.002	0.317
	Grade 5	1 (3.6%)	0		
Late esophageal stenosis	Grade 4	0 (0%)	1 (3.4%)	0.001	0.980
	Grade 5	1 (3.6%)	0 (0%)		
Pulmonary interstitial fibrosis	Grade 3	1 (3.6%)	1 (3.4%)	0.001	0.980

**Table 4:** Comparison of dose distribution parameters for tumor target volume and OAR between ENI Group and INI Group

Item	PTV volume (cm^3^)	GTV volume (cm^3^)	PTV Dmean (Gy)	GTV Dmean (Gy)	Lung volume (cm^3^)	MLD (Gy)	Lung V5 (%)	Lung V10 (%)	Lung V20 (%)	Lung V30 (%)	Thorax stomach Dmax (Gy)	Heart V30 (%)	SP Dmax (Gy)
ENI	353.5±123.7	47.0±24.1	49.9±2.6	60.2±2.5	3167±321	13.1±1.6	55.9±6.7	43.1±5.0	27.6±2.6	18.3±1.6	54.5±4.4	41.1±9.5	43.8±1.7
IFI	210±82.2	36.1±13.1	51.0±3.5	60.5±1.5	3224±471	10.8±0.9	48.7±4.2	37.9±3.7	19.9±2.4	12.0±2.1	55.9±4.3	38.0±8.8	42.4±2.7
F value	0.454	4.755	1.660	1.281	3.405	4.141	8.668	4.345	5.141	3.889	0.019	0.025	5.385
*P* value	0.014	0.038	0.203	0.268	0.07	0.049	0.005	0.042	0.018	0.035	0.890	0.876	0.024

Abbreviations: GTV, gross tumor volume; PTV, planning target volume; Dmean, mean dose; Dmax, absolute maximal dose; MLD, mean of lung dose; SP, spinal cord.
